# Time to re-set our thinking about airways disease: lessons from history, the resurgence of chronic bronchitis / PBB and modern concepts in microbiology

**DOI:** 10.3389/fped.2024.1391290

**Published:** 2024-06-07

**Authors:** Mark L. Everard, Kostas Priftis, Anastassios C. Koumbourlis, Michael D. Shields

**Affiliations:** ^1^ Division of Paediatrics & Child Health, University of Western Australia, Perth, WA, Australia; ^2^ Allergology and Pulmonology Unit, 3rd Paediatric Department, National and Kapodistrian University of Athens, Athens, Greece; ^3^ Division of Pulmonary & Sleep Medicine, George Washington University School of Medicine & Health Sciences, Washington, DC, United States; ^4^ Experimental Medicine, Queen’s University Belfast, Belfast, United Kingdom

**Keywords:** chronic bacterial bronchitis, airway disease, biofilms, microbiome, COPD, chronic bronchitis, bronchiectasis, protracted bacterial bronchitis (PBB)

## Abstract

In contrast to significant declines in deaths due to lung cancer and cardiac disease in Westernised countries, the mortality due to ‘chronic obstructive pulmonary disease’ (COPD) has minimally changed in recent decades while ‘the incidence of bronchiectasis’ is on the rise. The current focus on producing guidelines for these two airway ‘diseases’ has hindered progress in both treatment and prevention. The elephant in the room is that neither COPD nor bronchiectasis is a disease but rather a consequence of progressive untreated airway inflammation. To make this case, it is important to review the evolution of our understanding of airway disease and how a pathological appearance (bronchiectasis) and an arbitrary physiological marker of impaired airways (COPD) came to be labelled as ‘diseases’. Valuable insights into the natural history of airway disease can be obtained from the pre-antibiotic era. The dramatic impacts of antibiotics on the prevalence of significant airway disease, especially in childhood and early adult life, have largely been forgotten and will be revisited as will the misinterpretation of trials undertaken in those with chronic (bacterial) bronchitis. In the past decades, paediatricians have observed a progressive increase in what is termed ‘persistent bacterial bronchitis’ (PBB). This condition shares all the same characteristics as ‘chronic bronchitis’, which is prevalent in young children during the pre-antibiotic era. Additionally, the radiological appearance of bronchiectasis is once again becoming more common in children and, more recently, in adults. Adult physicians remain sceptical about the existence of PBB; however, in one study aimed at assessing the efficacy of antibiotics in adults with persistent symptoms, researchers discovered that the majority of patients exhibiting symptoms of PBB were already on long-term macrolides. In recent decades, there has been a growing recognition of the importance of the respiratory microbiome and an understanding of the ability of bacteria to persist in potentially hostile environments through strategies such as biofilms, intracellular communities, and persister bacteria. This is a challenging field that will likely require new approaches to diagnosis and treatment; however, it needs to be embraced if real progress is to be made.

## Introduction

It has been more than 200 years since Charles Badham used the term bronchitis to describe ‘an inflammatory affection of the mucus membrane which lines the bronchial tubes’ (1–3). This was not a new disease, but inventing a new name was a signal that the thinking about pulmonary disease was starting to better understand the implications of acute and chronic inflammation of the airways that accompanied most diseases of the ‘air sacs’ (alveoli) and pleura as well as being common in its own right. Well into the 21st century, chronic inflammation of the airway remains the cause of a substantial burden of morbidity and is amongst the highest causes of death worldwide (4, 5). Despite the intervening two centuries, progress in treating and preventing the consequences of ‘chronic bronchitis’ has been, at best, disappointing. In the middle of the 20th century, the current situation was summed up by Southwell who noted that ‘No one can pretend that the treatment of chronic bronchitis is anything but profoundly unsatisfactory’ (6) and Goodman who noted that ‘For many years this crippling disease (chronic bronchitis) has been complacently accepted (7). Seventy years later, a recent editorial in *The Lancet* (2022) noted that ‘Chronic obstructive pulmonary disease (COPD) has for too long been seen as a self-inflicted progressive disorder of smokers towards the end of life with few treatment options beyond symptom control. There has been no major progress in treatment or prevention for decades’ (8).

The author of that editorial also noted that the current situation, in which a ‘diagnosis of COPD is often accompanied by a sense of futility and a degree of stigma’ (8), should not be acceptable and that our goal in the C21st should be lifelong respiratory health for all. This comment appears to highlight the key issue issue holding back progress, which is the use of the term COPD to describe a disease when in fact it is an arbitrarily defined level of airflow obstruction resulting from chronic inflammatory airway disease. Much of the ‘sense of futility’ comes from the huge investment in guidelines for the management of ‘COPD’ with little to show in terms of impact on prevalence and mortality. Inevitably, if one starts in the wrong place, the results will be disappointing. The chronic inflammation responsible for airway damage is not only present in those who meet this arbitrary impairment of lung function. In most cases, it has predated a decline in lung function to this point by many years and indeed decades.

Chronic airway inflammation can cause dilatation in the larger airways that can be identified by the radiological appearance of bronchiectasis (no more a disease than COPD) on a CT scan. Similarly, in smaller airways, it can generally result in obstruction and fibrosis, which can lead to a degree of airway obstruction termed as ‘COPD’ when the FEV1/FVC is <70%, even if there is no change in the individual’s clinical condition. However, bronchiectasis and COPD are not diseases but just markers of the impact of chronic airway inflammation. Using these markers of damage to define ‘diseases’ is inappropriate and stifles understanding. Symptoms have generally been present for years or indeed decades before the appearance of these markers. An analogous situation to the current approach of producing discreet ‘COPD’ and ‘bronchiectasis’ guidelines would be for the cardiologists and public health teams to focus exclusively on producing guidelines for the management of an individual with myocardial infarction. The only nod to prevention would be smoking cessation while the management of hypertension might be included as a short-term intervention around the time of an acute event. Instead, prevention has been a key aspect of care involving the management of blood pressure, diet, and hyperlipidaemia, which has thrown up novel interventions such as statins that have an impact even in those who do not qualify to have ‘hyperlipidaemia’.

**Figure 1:**
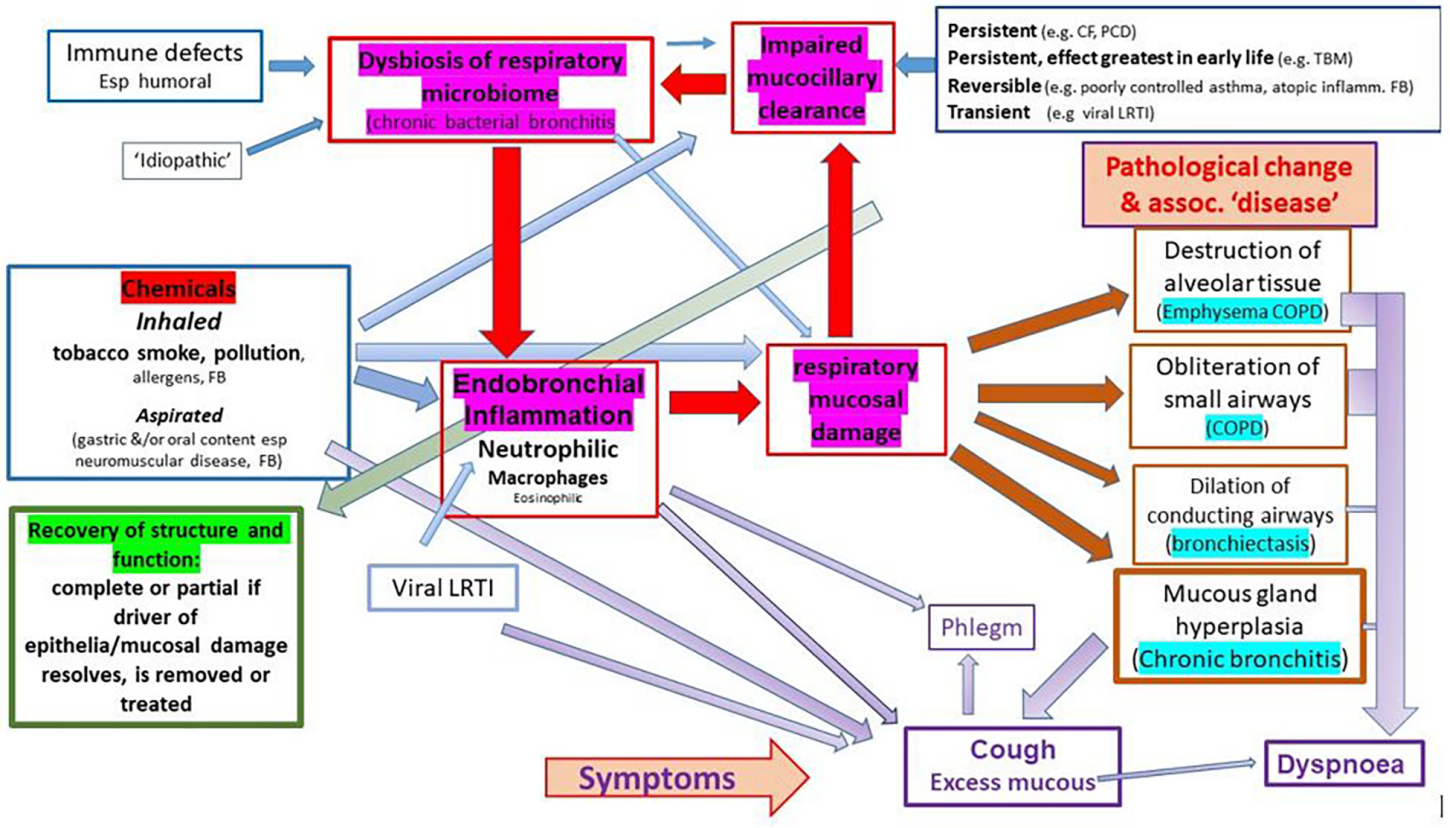
Drivers and consequences of airway inflammation and biofilm disease in particular.

We suggest, and argue in this paper, that airway diseases currently described as distinct and unrelated entities [e.g., ‘COPD’, ‘emphysema’, ‘chronic bronchitis’, ‘bronchiectasis’, ‘chronic suppurative lung disease’ (CSLD)] are a continuum resulting from chronic inflammation of the lower airways, with two great drivers of chronic inflammation being inhaled toxins (mostly due to inhaling the products of combustion in the form or cigarette smoking or indoor and outdoor pollutants) and chronic bacterial bronchitis (dysbiosis of the respiratory microbiome) (Figure 1). The latter is commonly superimposed on the former and often persists if the original driver is removed as in the individual who gives up smoking. The current siloed approach has effectively sidelined the role that chronic microbial-induced neutrophilic inflammation plays in the symptoms and progressive pathology experienced by many patients. It is unfortunate that, at a time when we are developing a much better understanding of what constitutes a chronic bacterial infection and the associated dysbiosis of the pulmonary microbiome, many are looking to deal with the problem through ‘anti-inflammatory’ approaches that do not address the underlying drivers of inflammation. To achieve improved respiratory health, we would argue that, in addition to reducing the burden of toxic agents inhaled by individuals through smoking cessation and addressing the many forms of atmospheric pollutants that challenge airway homeostasis, it is vital to develop effective treatment strategies that will deal with the highly developed defence mechanisms utilised by bacteria such as non-typable *Haemophilus influenzae* (NTHi) to persist in the face of threats such as antibiotics. Closer cooperation between respiratory clinicians and microbiologists who have an interest in bacteria strategies such as the formation of biofilms, intracellular bacterial communities (IBCs), persister bacteria, and disturbed microbiota offers the potential to take real strides towards prevention and mitigation of chronic bacterial infections of the airways. An important step in this process is to revisit our use of diagnostic labels, particularly that of COPD, which has helped hide the role of bacterial-driven neutrophilic airway disease in the symptoms and morbidity experienced by many patients.

Two hundred years ago, Andral, in his preface to the Fourth Edition of Laennec's remarkable text (9), noted that ‘Medical history is replete with the mistakes of those who do not wish to learn for themselves; those who ignore the past; and those who accept doctrines without critical examination’. In this review, we aim to highlight the important lessons of the past and challenge the currently accepted doctrines while drawing on the lessons learnt about chronic bacterial bronchitis in children during the past decades.

### Lessons from history

This section will contain a summary of the evolution of our thinking regarding airway disease over the past 200 years.

### Most of the pathology had been described by the mid-19th century, and this included an understanding of the impact of inflammation on different regions of the conducting airways and respiratory zone

It is important to recognise that the term ‘bronchitis’ was used by Badham to describe an inflammation of the conduction airways, which include both the bronchi and bronchioles. The conducting airways are a continuum, and, hence, it is inconceivable that inflammation is limited to just the larger bronchi or the smaller distal bronchioles. At around the same time, Laennec published his great work, which included detailed descriptions of bronchiectasis, obliteration of small airways (bronchioles), and emphysema amongst other pathologies (9). He noted that these three types of pathologies commonly coexist and that bronchial catarrh (mucoid/purulent secretions) were almost universally present. He was clear that the same process could produce different pathological changes depending on the size and structure of the affected airway. The term chronic bronchitis was well accepted by the time Stokes published his textbook *Diagnosis and Treatment of Diseases of the Chest* in 1837 (10). He considered bronchitis to be the most important disease of the lungs, noting ‘we find that bronchitis is present and has a most important share in almost all diseases of the lungs, whether acute or chronic’. He, as did other authors, noted that acute bronchitis was generally most severe in the young and that chronic bronchitis generally resulted from unresolved acute bronchitis. In adults, acute bronchitis was most problematic in those with chronic respiratory symptoms that manifest as episodes of more severe symptoms that could be fatal on a background of chronic cough and sputum production. He noted that a patient's secretions (catarrh) could range from transparent mucous to puriform and could change overtime. Many appeared relatively well despite cough and expectoration, which frequently largely abated in the summer becoming more troublesome again in the winter. With time, the relative remissions diminish, and the cough and sputum become permanent. He noted that such a natural progression generally ended in ‘dilation of the tubes’.

Crucially, Stokes clearly understood the different susceptibilities of the conducting airways with mucous gland hypertrophy and dilation of larger cartilaginous bronchi (bronchiectasis) with obliteration of the smaller ‘non-cartilage baring bronchi’ or bronchioles (obliterative bronchiolitis) and associated dilation of ‘air sacs’ (emphysema), again noting that these findings were not exclusive but commonly found together. Obliteration of the larger airways was also noted on occasions, but this was far less common than bronchiectasis. These observations were again made in the 1950s by L. Reid and others in their studies on patients with chronic bronchitis and ‘bronchiectasis’ (11–17) but were all too often forgotten. As noted by Walshe in his 1851 textbook (18), the term ‘exacerbation’ was already in use for a severe ‘acute attack’, and he noted that the link between disease activity and general wellbeing noting that he has known ‘as much weight lost during the first three weeks of an annual recurrence of chronic bronchitis as in the same period of cases of consumption in active progress’.

It should be noted that these observations were made long before the era during which cigarette smoking became common. Infections and pollution (indoor and outdoor) largely due to burning coal were the dominant drivers of these ubiquitous airway diseases (3). The link between loss of airway smooth muscle homeostasis and asthma (18–20) had also been made by this time accurately marking out asthma as a separate disease from those being considered in this review (although poorly controlled asthma certainly predisposes to the development of a persistent bacterial bronchitis). Walshe (18) also highlighted the link between inhaled allergens such as grass pollen with hay fever and seasonal asthma.

### Germ theory, pneumonia, and outcomes

While morbid anatomy and histology had greatly advanced our understanding of the consequences of disease, clinicians were still struggling to understand the causes of diseases and how to treat them. The idea that ‘germs’ could cause disease was not invented in the second half of the 18th century but gained strength and eventually became irresistible during this period through the works of many scientists including Pasteur and Koch (21, 22). *Streptococcus pneumoniae* (Str Pn) was identified in1881 and had conclusively been linked with lobar (alveolar) pneumonia within a few years (23).

It is of note that studies from the early 20th century, which is in the pre-antibiotic period, reported that ‘bronchopneumonia’ was significantly more common than alveolar pneumonia and had a significantly greater mortality. In a large paediatric study, the mortality from bronchopneumonia was 54% vs. 7% in those with an ‘alveolar’ pneumonia (24). The term alveolar pneumonia was used rather than lobar pneumonia as it frequently did not (and still does not) affect the whole lobe.

Mortality in those with empyema was reported at 40.5%. Most deaths in this era were in the very young and those in later life (3, 24). In another study, considering the impact of weather on deaths under 5 years of age, it was noted that death rates due to ‘bronchitis’ were significantly higher than those due to pneumonia (25). Presumably, both studies included many infants with what we would now describe as having acute bronchiolitis (26) while the high rate of deaths in the older population was attributed to exacerbations of chronic bronchitis (3). However, it is important to remember that Str Pn infections are a particular problem in infants (23) while a study published in the pre-antibiotic found that *Haemophilus influenzae* (HI) was isolated from the lower airways of 95% of children dying from bronchopneumonia (27).

The distinction between lobar (alveolar) and bronchopneumonia is often blurred, but publications from this era suggest that for those with pneumonia developing an acute febrile illness on the background of being well, most commonly had ‘alveolar’ pneumonia with organisms spreading rapidly locally through the alveoli and alveolar ducts with little bronchial involvement. Such illnesses would commonly terminate with a ‘crisis’ leading to recovery or death. In those with chronic bacterial bronchitis, exacerbations are not uncommonly associated with patch chest x-ray (CXR) changes. The illness is frequently less acute but more indolent and, in the pre-antibiotic era at least, had a higher mortality. In this era, HI had not been classified, and many such deaths are likely to have been due to NTHi. However, as with much of respiratory medicine, distinctions are often blurred.

Currently, the vast majority of children with pneumonia who are treated with antibiotics (usually orally) are expected to make a full recovery. However, a systematic review found high levels of morbidity following a diagnosis of ‘pneumonia’ when studies published between 1970 and 2011 (well into the antibiotic era) were considered (28). Of those hospitalised without a pathogen being identified, 17.6% had evidence of restrictive lung disease, and 4.2% developed chronic bronchitis. Few follow-up studies have considered chronic respiratory symptoms, but in one that did, the incidence of chronic cough in children with radiologically proven pneumonia was three times that in the control population (29).

In the pre-antibiotic era, levels of respiratory morbidity following lower respiratory tract infections were much higher (30, 31). A follow-up study of children admitted with an illness consistent with pneumonia found that 25% were still experiencing chronic respiratory symptoms consistent with ‘chronic bronchitis’ some years later (30). In a Canadian study of army personnel with radiologically proven pneumonic illness, Andrus (32) noted that patients generally recovered rapidly with CXR changes resolving by 3 weeks or developed a cough and other symptoms, which might take some time to resolve or fail to resolve at all. Respiratory illnesses were common in this population due to their confined living conditions in barracks. Those with ongoing symptoms usually had residual CXR changes, and he noted that ‘Such changes which are variously named chronic infectious basal disease, chronic nontuberculous disease, chronic post-pneumonic disease or injury, chronic pneumonitis, pulmonectasis, etc. may be of any intensity, from slight to gross’. Highlighting the numerous terms used to describe a given condition, he also noted that bronchiectasis may or may not be present if sought in such patients and that its presence was not predictable by clinical or radiological changes. Chronic non-tuberculous disease was the most common reason for medical discharge from the army.

The historical evidence from these and other studies indicates that a significant proportion of those who had a pneumonic illness but survived in pre-antibiotic days had ongoing respiratory symptoms, and this was an issue in both children and healthy adults. It was also known that whooping cough and measles were two other precursors to chronic symptoms (with some developing radiological changes of bronchiectasis). However, in most cases irrespective of age, ‘chronic bronchitis’ (cough and sputum) was considered to be a consequence of unresolved acute bronchitis (6).

### Germ theory and chronic bacterial bronchitis

In the early years of the 20th century, the ‘usual suspects’ [Str Pn, HI, *Moraxella catarrhalis* (MC)] that are still primarily associated with chronic bacterial bronchitis had been identified in those with chronic airway diseases (33–35). *Pseudomonas aeruginosa* was not linked to CF and other advanced respiratory diseases until half a century later (36). Reports of using vaccines prepared from sputum containing these organisms to treat those with chronic bronchitis and asthma appeared before the First World War (37, 38), and this approach has become a recurring theme in the subsequent 100 years (32, 39–43). The interest in vaccines developing so soon after the link between chronic bronchitis and bacteria had been established is understandable in that there were no effective treatments (the development of antibiotics was decades in the future) and there was a history of vaccines being able to prevent disease going back to smallpox vaccines. In the case of those with chronic bronchitis, it was hoped that the vaccines might also help treat those affected. A number of reports, but not all, suggested some efficacy. The hope that chronic bacterial respiratory disease can be treated with ‘vaccines’ persists to this day although with little success to date. A further flurry of studies in the 1950s confirmed the importance of these pathogens in chronic bronchitis (44–49), and a regular procession of studies since (50, 51) then have all confirmed the observations made more than a hundred years ago.

### Bronchiectasis becomes a ‘disease’, but it is recognised that ‘chronic bronchitis’ and ‘bronchiectasis’ generally cannot be distinguished clinically

Many authorities in the late 19th and early 20th centuries commented on the difficulty in identifying patients with bronchiectasis in life. They noted that in advanced ‘classical’ cases, the patients appeared unwell, produced copious purulent secretions, were wasted and clubbing, and had a very poor prognosis. These patients generally died of bronchopneumonia, ‘exhaustion’, toxaemia, massive haemoptysis, or cerebral abscess. However, it was clear from postmortem examinations that many had much milder disease with symptoms indistinguishable from the majority of patients with chronic bronchitis and could be present in the absence of any symptoms. While the duration between apparent onset and death could be relatively short, in others, the development of bronchiectasis was considered to be associated with a chronic disease that might cause symptoms for decades (52–58). A 1920 report noted that 2% of admissions to Brompton Hospital were reported to have bronchiectasis, but this was probably a gross underestimate as it was always a consequence of other disease processes (53). Chronic bronchitis, unresolved pneumonia, whooping cough, measles, and airway obstruction by a tumour or foreign body were reported as by far the most common causes in those without tuberculosis. McNeil and colleagues reviewing the field in 1929 noted that ‘From all these data, it would seem that the majority (probably the great majority) of cases of bronchiectasis date back to early childhood and originate in broncho-pneumonia or bronchitis’ (55).

The advent of bronchograms in the 1920s led to a renewed interest in this form of pathological change (55, 59–74) as doctors feel much more comfortable when they have a test rather than having to rely on their clinical acumen. Having this test completed, the term ‘bronchiectasis’ moved from a pathological description to becoming a ‘disease’ even though it was universally considered to be the visible consequence of another disease. Many bronchograms were carried out to produce a ‘definitive’ diagnosis, but the results had real clinical consequences. In the absence of effective treatments other than postural drainage and a change of climate, surgical resection of affected lobes in those with particularly problematic symptoms became the treatment of choice. Bronchograms were required to identify diseased areas of the lungs that could be resected.

As the use of bronchography increased, a number of features of chronic respiratory disease were clarified, and by the 1950s, it was clear that:

a) ‘Dilation of the bronchi does not always produce signs or symptoms’ (54). ‘Dry’ bronchiectasis was well recognised and often diagnosed in asymptomatic patients presenting with haemoptysis (54, 57, 62)
b) Clinical symptoms alone could not distinguish between those with and without bronchiectasis. Many patients believed to have bronchiectasis had negative bronchograms (75) while bronchiectasis in those with simple ‘chronic bronchitis’ was not uncommon. In a study of service personnel undertaken at a military hospital in which bronchograms were performed on 214 service personnel referred with ‘symptoms and signs suggestive of chronic bronchitis’, 46 (21%) proved to have evidence of bronchiectasis when a bronchogram was undertaken (68). The authors noted that such patients had often been labelled as having ‘simple bronchiectasis’ by other authors and note that ‘Such cases are rarely seen in civilian hospitals, because under ordinary conditions they have no difficulty in carrying on their work, the annual bouts of winter bronchitis being regarded as inevitable and lightly dismissed’.
c) Studies demonstrated that children and adults with symptomatic chronic bronchitis and negative bronchograms might develop unequivocal bronchiectasis when re-examined a few years later giving rise to the term pre-bronchiectasis as an alternative to chronic bronchitis (70, 72, 76). In one follow-up study undertaken prior to the antibiotic era, 40% of children had ‘pre-bronchiectasis’ based on having symptoms but were found to have developed bronchiectasis 3 years later while 37% were ‘cured’ or rather had become symptom-free (70). However, while a valuable concept highlights the opportunity for prevention, pre-bronchiectasis can only be applied accurately in retrospect.
d) Bronchiectasis was commonly localised to certain lobes or segments of a lobe, especially in early disease. Changes were not static, and over time, other lobes in both the ipsilateral and contralateral lung frequently became involved. This suggests that atmospheric pollution alone was unlikely to be responsible given that deposition would be widespread; rather, it strongly favours bacterial infection as the primary driver of the damaging inflammation, although persistent and/or recurrent infection in a given area was likely to have been secondary to causes of impaired mucociliary clearance.
e) Bronchiectasis was not necessarily ‘irreversible’ with reports of it resolving in adults and children at least in the early stages (52, 70, 77–79), and such reports continue to appear (80, 81).
f) The majority of those affected appeared to have first developed symptoms in childhood most commonly in the first 2 years of life (55, 66, 82, 83). A community-based study noted that 44% of those identified with bronchiectasis had been symptomatic before the age of 10 years but most had not come under medical until their fourth decade with a median delay between the onset of symptoms and referral to secondary care of more than 17 years (83). Even in the post-antibiotic era, studies are producing similar results (84).
g) Typically, it followed chronic symptoms persisting after acute lower respiratory tract infections such as acute bronchitis, pneumonia, whooping cough, measles, and TB (10, 52–90). It should be noted that in 1947, the most common cause for death amongst infants after congenital abnormalities and prematurity were ‘respiratory diseases’, and for those aged 1–4 years, the top 5 were pneumonia, TB, violence, whooping cough, and measles (91)
h) Surgical resection of affected lobes remained the treatment of choice for many well into the antibiotic era (92).
i) Follow-up studies of children with significant bronchiectasis indicated that the majority improved clinically in their teens and twenties with deterioration being uncommon (93, 94). Longer-term follow-ups into middle age were not pursued, but poor recall of childhood events likely accounts for many cases of ‘idiopathic’ bronchiectasis presenting in middle age (83). There are few recent follow-up studies. One follow-up study involved the indigenous children in Alaska who had developed bronchiectasis in early childhood and found high levels of morbidity in adults aged 20–40 years who had poor transition of care, with only 23% being asymptomatic. The majority of patients and their physicians were not aware of their diagnosis despite the high levels of respiratory morbidity (95).
j) In terms of pathology, there have been ongoing debates about the processes leading to dilation of the medium to large bronchi and obliteration of bronchioles. From Laennec (9) onwards, inflammation associated with chronic pulmonary catarrh or chronic bronchitis was believed to be a key component, although by the end of the 19th century, ‘traction’ due to adjacent collapse was also considered by some to be a factor in many cases. Bacterial infection driving inflammation was implicated in the early 20th century, and the idea of a vicious circle of impaired clearance, infection inflammation, and damage was commonly held to be a key component by the mid-20th century (96, 97) recognising that the circle of infection, inflammation, damage predisposing to infection applied to chronic bronchitis and did not necessarily result in bronchiectasis. One publication from the 1950s noted that ‘Nowadays the divergence of opinion seems to be occupational, for, while many physicians and radiologists think of the bronchial inflammatory changes as mild and the dilatations of a mechanical nature, most surgeons and pathologists regard bronchiectasis as a destructive inflammatory process’ (98)*.* The later, like Laennec, presumably had the advantage of seeing the condition in front of them while the physicians and radiologists could only speculate from afar. In earlier publications, the existence of a vicious circle was assumed to be involved in the pathogenesis of chronic bacterial bronchitis and could lead to bronchiectasis in some. Following later iterations of the concept, the ‘vicious circle’ became linked to ‘bronchiectasis’ (99, 100), and the importance of this cycle prior to the development of bronchiectasis and ‘fibrosis’/obliteration of bronchioles came to be forgotten although this was never the intention of Cole.

The relatively large number of papers regarding bronchiectasis contrasted with the relative lack of interest in chronic bronchitis illustrating the impact of a ‘test’ on priorities and medical thinking. Those with bronchiectasis might benefit from surgery, and those without were offered cough medicine until such time they developed bronchiectasis. This seems to sum up current approaches to management and prevention if one changes antibiotics for surgery.

### Antibiotics and chronic bronchitis' moment in the sun

The great step change in improving respiratory health that took place in the mid-20th century was the development of antibiotics (101, 102). Finally, clinicians had an opportunity to effectively treat the bacterial pathogens that knew so well but about which they could do little. The extraordinary impact of these ‘magic bullets’ is often forgotten. Their impact on treating TB and acute life-threatening infections including pneumonia was nothing less than miraculous. An illustration of the dramatic impact of sulphonamides, penicillin, and other antibiotics on the survival of children with pneumonia and the prevention of progression to bronchopneumonia and chronic symptoms was contained in a 1952 publication from Milwaukee (103). The increasingly widespread use of antibiotics over the following decades had a dramatic effect on the incidence of chronic bronchitis in children, which in the 1940s had been described as a very common condition (104), to the point where few respiratory physicians believed it existed. Antibiotics also transformed the lives of children with bronchiectasis leading to dramatic declines in the number of admissions to children's hospitals, in some cases 10-fold reductions in just a few years. As noted by Kassowitz et al. (103) as early as 1952, not only did the number of admissions with bronchiectasis decline dramatically as did its apparent incidence (98). Within a couple of decades, the incidence had fallen so far that most paediatric and adult physicians considered it to be an ‘orphan disease’ encountered rarely and of little importance in the overall scheme of health-related problems (105, 106). This decline in incidence and prevalence was not because of the effect of antibiotics on those with bronchiectasis but almost certainly due to their effect on the health of individuals given antibiotics for acute bronchitis—the widespread use of antibiotics in primary care likely prevented many with viral bronchitis and pneumonia developing superadded persistent or chronic bacterial bronchitis, which was the route into chronic poor respiratory health for many patients.

The hope that antibiotics would transform the lives of those with chronic bronchitis, given the known ubiquitous presence of bacterial pathogens in sputum, suddenly provided clinicians with the hope that they would have an effective therapy that went beyond a bottle of cough medicine. This led to a renewed interest in the microbiology associated with chronic and recurrent airway disease, which confirmed earlier work regarding the importance of the ‘usual suspects’ and in particular that of Strep Pn and HI (44–49), The idea that HI was identified in up to 90% of sputum samples from those with chronic airway disease led to further trials of HI vaccines but seemed to have little impact (107). Hers and Mulder (108) demonstrated that HI, unlike viruses such as influenza and measles, did not cause significant epithelial damage and was often identified between epithelial cells or even below the epithelial layer. They also noted that despite the lack of evident damage caused by the bacteria, there was an associated inflammatory response. They speculated that viruses provided the opportunity for HI to establish a chronic colonisation/infection.

Early studies noted the beneficial effects of short- and long-term antibiotics when used to treat the chronic symptoms of both bronchiectasis and chronic bronchitis and prevent and treat exacerbations (31, 109–122). Investigators noted that it took 2 weeks or longer for a significant improvement to be observed and that if there was no improvement by 4 weeks, therapy should be discontinued, which is very similar to recent observations in children with persistent bacterial bronchitis (PBB). However, it soon became apparent that recurrence of chronic symptoms and exacerbations were all too common on discontinuing the antibiotics, especially amongst those with NTHi (31, 109, 110, 114, 116, 117, 123), with the suggestion that the organism was able to persist despite antibiotic therapy (123, 124) although investigators were uncertain of the mechanism. The idea of dormant ‘persister’ bacteria was well established in 1944 (125), decades before the recognition of the role of biofilms and IBCs in human diseases (126–131). The recognition that acute illnesses generally involve more virulent rapidly dividing planktonic bacteria and that chronic infection with biofilms and occasional flare-up with the release of planktonic bacteria is still in its infancy in respiratory medicine.

Despite the potential for benefit, the push back against the use of antibiotics in chronic bronchitis started commenced at an early stage. The realisation that in many/most cases the patient would not be cured led, understandably, to concerns regarding the potential impact on antibiotic resistance of repeated or long-term prescribing and suggestions that the cost of treating such a common chronic disease in this way would be too high for the fledgling health service (132, 133). It is perhaps worth noting that early studies found little resistance developing in the patients. This is similar to more recent experience with PBB when the same antibiotic can often be used for prolonged periods without apparently losing its effectiveness. Studies from this period have subsequently been misinterpreted as suggesting that antibiotics do not have a role in the treatment of patients with chronic bronchitis/COPD other than brief exposure to antibiotics during ‘exacerbations’ re-reinforcing the perception that infection is not an important component. For example, the Global Initiative for Obstructive Lung Disease (GOLD) reports suggest that antibiotics for chronic bronchitis did not work based on three studies, which, when published, argued in favour of a positive effect (120–122) with a conclusion directly in opposition to those suggested by GOLD. For example, in one study that reported that antibiotics had no impact, a 50% reduction in days lost from work was observed (120). A modern drug with such an impact would be hailed as a major breakthrough and promoted aggressively by the relevant pharma company.

These studies indicated that antibiotic therapy can make an impact but they were limited by factors such as failing to understand the nature of biofilm diseases, including smokers in whom the benefit would be limited by the ongoing inhalation of toxins (a greater effect is likely to be seen in ex-smokers), and the need to achieve high concentrations in the disease airways. Unfortunately, their results are still being misinterpreted as implying that antibiotics have no effect.

### Other significant changes in risk factors for poor respiratory health

Physicians had long recognised that environmental pollution, along with bacterial infections, was an important driver of poor respiratory health. Atmospheric pollution in the industrialised cities of the UK peaked in the 1880s and had fallen significantly over the subsequent 60 years (3, 134, 135). The Great London Smog of December 1952, which killed over 4,000 individuals in 4 days (136) and led to a long tail of excess deaths and increased morbidity, was the last of the notorious London smogs. This progressive decline in pollution, mostly from the combustion of coal, was paralleled by a significant and progressive decline in deaths due to acute and chronic bronchitis, at least until the 1940s (3). This trend was in direct contrast to the progressive rise in the consumption of cigarettes that started at the end of the 19th century with the invention of machines that could produce large quantities of cheap cigarettes (137). The rise in smoking rates was accompanied by a parallel rise in lung cancer rates although this lagged approximately 20 years behind smoking rates (3, 138). Other factors, such as vaccination against a variety of diseases including measles and whooping cough, improved housing and living standards, and reduced overcrowding, all helped improve respiratory health after the Second World War. Unfortunately, the inexorable rise in smoking rates through the 20th century continued until it peaked at around 70% of men in 1960. A focus on smoking cessation came to the fore when it was shown that cigarette smoking was closely linked to major causes of mortality, such as lung cancer, ischaemic heart disease, and stroke. This focus on smoking, the enormous impact of antibiotics on the visibility of chronic bronchitis, and the invention of the ‘disease’ COPD contributed to clinicians losing sight of the importance of chronic bacterial bronchitis, which has become the elephant in the room.

### Use and misuse of diagnostic labels

As the 1950s gave way to the 1960s and hopes that antibiotics would rapidly cure those with chronic bronchitis were fading, the issue of diagnostic labels became more topical. The intention was to improve diagnostic criteria and accuracy, which would in turn help patients, improve the data generated in epidemiological studies, and improve therapy. A number of studies demonstrated that then, as now, misdiagnosis of airway disease was rife such that a ‘doctor’s diagnosis’ of asthma or chronic bronchitis was commonly found to be incorrect when a more detailed examination of a patient was undertaken. A doctor was expected to recognise a disease when he saw it but had little to guide his perception. Similarly, it was believed that one reason for the high prevalence of chronic bronchitis in the UK (the English disease) when compared with other countries was the differences in diagnostic labels fashionable at the time (139–141). This was summed up in the introduction to the CIBA meeting that attempted to improve the situation, a summary of which was published in 1959. ‘At present the diagnoses “chronic bronchitis,” “asthma,” and “emphysema” are used without any general agreement about the clinical conditions to which they refer. Anyone (or more) of these words may be used by different clinicians to describe the condition of the same patient. It appears that chronic bronchitis is often used in Great Britain to describe cases that would be called asthma or emphysema in the United States’ (139).

By the late 1950s, the field of respiratory medicine changed rapidly with the advent of a number of effective therapies. The first pressurised meter dose inhaler (pMDI) containing adrenaline replaced the glass handheld single-dose nebulisers containing adrenaline for the treatment of asthmatic bronchospasm (142), the effectiveness of oral steroids in the treatment of asthma was recognised (143), and, as noted above, antibiotics were shown to influence on the morbidity experienced by those with bronchiectasis and chronic bronchitis. The CIBA symposium aimed to produce robust clinical definitions that would help improve diagnostic accuracy and ensure patients would be prescribed the most appropriate treatment.

Their recommendations included the recommendation that the conditions being addressed formed a group of chronic non-specific lung diseases, which manifest as (1) chronic or episodic excessive secretion of bronchial mucus (*chronic bronchitis*), (2) intermittent obstruction to bronchial airflow (*asthma*), or (3) persistent obstruction of bronchial air flow *(irreversible or persistent obstructive lung disease*) (139).

Chronic bronchitis should be diagnosed clinically based on a history of a ‘chronic or recurrent cough with expectoration which is not attributable to conditions excluded from chronic non-specific lung disease. Infection of the bronchi is frequently but not necessarily present. Not infrequently subjects who produce sputum deny cough. Such subjects are included as having bronchitis. Subjects who habitually swallow sputum should also be included as having chronic bronchitis’. Thus, they were fully aware of key reasons that the condition frequently goes unrecognised and that there were no artificial time criteria. Excluded conditions included ‘localised lung diseases’ such as tuberculosis, pneumonia, and ‘bronchiectasis’ and other conditions such as pneumoconiosis, collagen disease, and psychoneurosis. Scadding (141), at around the same time, agreed with this definition, which does not aim to consider aetiology and is also notable for not having a minimum duration. He was influenced by the histological studies of Reid and the recognition that dyspnoea was a late and non-inevitable feature. He suggested this definition be used for the earlier ‘non-disabling phases’ with the term ‘chronic bronchitis with pulmonary insufficiency’ used to label the disease of those with chronic bronchitis who had, over time, developed a significant functional impairment of the respiratory system. In discussing the ‘jocular’ terms ‘pink puffer’ and ‘blue bloater’ that had crept into the UK medical terminology, he notes that the development of dyspnoea attributed to emphysema without pre-existing chronic bronchitis was very uncommon (141).

The CIBA Foundation report noted that ‘Asthma refers to the condition of subjects with widespread narrowing of the bronchial airways, which changes its severity over short periods of time either spontaneously or under treatment, and is not due to cardiovascular disease’. This definition has stood the test of time and remains the basis of objective diagnosis. In many countries, the lack of freely available spirometry in primary care has contributed to the ongoing high prevalence of over- and underdiagnosis of asthma, which is a defect that is only slowly being addressed. Physicians would request a full blood count if they are concerned about anaemia but are happy to hand out an inhaler in the absence of an objective disease confirmation. Thus, asthma was defined as a disorder of function, which may be exacerbated by factors such as allergies, which were neither sufficient nor necessary.

The participants of the CIBA Foundation Symposium recognised that more than one feature could be present in an individual patient and deliberately chose the term irreversible obstructive lung disease recognising that this would include patients with both emphysema and loss of bronchioles should their lungs be examined histologically.

Cognisant of the vagaries of clinical acumen, they also stated that lung function testing was essential and should include an assessment of bronchodilator responsiveness (139, 144). Doctors like to have a test and feel on more certain ground when they have a test that will help make the diagnosis—few would make a firm diagnosis of anaemia (unless extreme) without having obtained a full blood count assessment. However, tests are not always enlightening. The adoption of bronchography provided doctors with confidence that a structural change had occurred in the airways of patients with chronic bacterial bronchitis, but the adoption of artificial criteria for a new disease, ‘bronchiectasis’, masked the fact that this was a marker of damage, not the underlying disease, and did not necessarily correlate with disease activity or morbidity.

Unfortunately, the same error was repeated when impaired lung function was labelled a disease rather than a descriptor of the result of a disease process. The term COPD was coined in the USA in 1967 (2) and slowly came to dominate the literature relating to chronic disease of the airways excluding ‘bronchiectasis’, as so often by the self-interest of pharma. The 2021 GOLD report noted that ‘Spirometry is required to make the diagnosis and that a post-bronchodilator FEV1/FVC <0.7 confirms the presence of persistent airflow obstruction’ (5). Again, a test has hidden more than it has revealed. This focus on an arbitrary lung function-defined cut-off meant that, as with ‘bronchiectasis’, symptomatic patients who had not demonstrated sufficient deterioration in lung function (or evidence of dilated bronchi on CT scans) are largely ignored despite symptoms that are frequently indistinguishable or worse than those with either of these two ‘diseases’ defined by an all or nothing test result. It is recognised that anaemia is a relatively late feature of iron deficiency anaemia, but this does not prevent treatment of iron deficiency if identified and a search for the driver of that deficiency to prevent anaemia from developing. This reliance on lung function and imaging to make an artificial clinical diagnosis has acted to obscure the disease process leading to the structural damage identified in the ‘test’. Once sufficient damage has been caused and identified this ‘permits’ a clinician to become more proactive in their management as the patient then fits into a ‘guideline’. The ‘treatment’ options are borrowed from ‘reversible’ airway obstruction (asthma) along with non-pharmaceutical interventions such as ‘pulmonary rehabilitation’ and smoking cessation programmes. Unfortunately, these have not been shown to have a significant impact on long-term outcomes and, other than smoking cessation, have no role or impact on the progression of the disease.

### ‘Chronic bronchitis’ becomes the Cinderella of airway disease in part because it did not have its own ‘test’ and because of well-intentioned but misguided attempts to improve the epidemiology

As noted above, the CIBA Foundation meeting generated a perfectly usable clinical definition of chronic bronchitis. A few years later, the Committee on Diagnostic Standards for Nontuberculous Respiratory Diseases of the American Thoracic Society (145) came up with definitions similar to that generated at the CIBA Foundation Symposium *but* introduced a time constraint on the duration of symptoms prior to making a diagnosis of chronic bronchitis producing a definition that is widely quoted today—patients were required to have a ‘chronic productive cough for at least 3 months per year in two consecutive years’. A British Medical Research Council that attempted to define chronic bronchitis in 1965 (146) noted that ‘simple chronic bronchitis is defined as chronic or recurrent increase in the volume of mucoid bronchial secretion sufficient to cause expectoration’. Again, they noted swallowing sputum habitually (a habit adopted by many, especially women) equated to expectoration. To compare *epidemiological studies*, the phrase chronic or recurrent should imply ‘expectoration has occurred in most days during at least three consecutive months for more than two successive years’. The aim of this more stringent definition was presumably to try and limit inclusions to those who unequivocally had persistent symptoms for significant periods. Using this ‘definition’, a patient was only deemed to have developed ‘chronic bronchitis’ after a minimum of 15 months, and indeed, in theory, they may have to wait 24 months for a diagnosis should they have two episodes separated by 18 months. This definition was not intended for clinical use. The current misuse of ‘diagnostic’ labels is summarized in Table 1.

**Table 1:** Current use and misuse of terms used to label airway disease.

Medical term	Intended use	Current use
Bronchiectasis	**Pathological description** Can be observed on a CT scan, bronchogram, or pathological specimen. Usually resulting from chronic inflammation and accompanied by loss of small airways	A ‘disease’ even though an individual may be entirely symptom-free Antibiotics used freely and for prolonged periods
‘COPD’	**Physiological criteria** Non-reversible airflow limitation based on an arbitrary value of FEV1/FVC at <70% predicted. May have been present since infancy or developed in the eighth decade	A ‘disease’ even though a patient may report few if any symptoms. Erroneously became synonymous with smoking cigarettes Antibiotic use limited to short periods during significant exacerbations
Chronic bronchitis	**Pathological term** Indicating chronic inflammation of the airways. Usually manifest by chronic cough and sputum production, which may range from minimal to major disruptors of quality of life	A ‘disease’ characterised by chronic /recurrent productive cough for >3 months in two consecutive years. Definition initially intended for use in epidemiology not for clinical purposes Symptoms often ignored or left untreated
Emphysema	**Pathological description** Disruption of the respiratory region usually accompanied by loss of small airways	Often used as a synonym for COPD
Chronic bacterial bronchitis (Persistent bacterial bronchitis)	**Bacterial-driven neutrophilic inflammation** Certain bacteria able to persist in excessive quantities through the use of biofilms and other strategies disrupting the normal health microbiota and homeostasis of the airways	Rarely used for adult patients Currently no consistency in diagnosis or treatment, which is often influenced by perceived ‘disease’ such as ‘bronchiectasis’, ‘COPD’ or ‘chronic bronchitis’, and ‘PBB’

### The impact of antibiotics through the second half of the 20th century

In 1963, a panel discussion regarding the role of antibiotics took place and was reported on in *The BMJ* (147). Towards the end, the chairman asked ‘Apart from clearing up the atmosphere and cutting down smoking, is there anything else we can do?’ to which Dr. Watson (a GP) replied ‘I think so. I like to believe that the prompt curative antibacterial treatment of acute chest infections in adults and children is equivalent to prophylaxis against chronic bronchitis in the next generation’.

Although this was never subject to scientific study, the rapid fall in the incidence of bronchiectasis and chronic bronchitis in developed countries outside of the setting of cigarette smoking over the following decades pointed to Dr. Watson's hope being realised. This was an era of widespread use of antibiotics for many conditions including ‘chest infections’. While this practice has been criticised for helping to drive antibiotic resistance, it was driven in large part by the feedback clinicians received from patients. However, this practice may also have significantly reduced antibiotic exposure in millions of individuals by preventing the establishment of a chronic airway disease (not only ChrBr but also chronic otitis media and chronic sinusitis). As noted above, identifying bronchiectasis became uncommon to the point that clinicians described it as an ‘orphan disease’ while ‘chronic bronchitis’ became a minor issue dealt with in COPD guidelines as an irritating minor comorbidity.

### Resurgence of chronic bronchitis and bronchiectasis—going around in a circle

In the past two decades, these advances have gone into reverse, and we find ourselves going around in circles (148). Perhaps inevitably this was first noted in young children with a number of groups reporting a resurgence of chronic bronchitis in the first part of the 21st century (76, 149–163). A new name of protracted bacterial bronchitis (PBB) was coined for this oldest of diseases, and it has now taken its place amongst ‘cough’ guidelines around the world (164, 165). For many years, many paediatric respiratory physicians did not accept that chronic bacterial bronchitis could occur in children, and the concept has still not filtered down into secondary and primary care to any significant extent. This is a major obstacle to prevention and early treatment. There are a few recent reports of adults with similar presentation (wet cough responding to antibiotics!), but it is almost certainly being significantly under-recognised in adults. More recently, bronchiectasis has again come to the fore with adult services for this group being expanded, and it is no longer viewed as a rare disease (166). The delay in recognition of the progressive increase in bacterial-related airway disease when compared to that seen in paediatric services is perhaps inevitable.

### Lessons from ‘PBB’

Prior to the 1960s, clinicians recognised that chronic bronchitis was a very common childhood condition, but in the 1980s, the idea that children might develop chronic bronchitis (in the absence of a condition such as CF or immunodeficiency) was denied by the majority of paediatric respiratory physicians even though a significant number of epidemiological studies from a range of countries reported a significant percentage (often >10%) of children with chronic cough with phlegm (167).

In the following decades, a number of reports started to appear suggesting that such patients were again becoming relatively common, and in 2006, one group coined a new term protracted bacterial bronchitis ‘PBB’ to describe such patients (155, 156). What distinguished this ‘new disease’ was that the label could be applied after only a few weeks (4–8 weeks although most were being referred much later) of ongoing wet cough, a return to the original descriptions of chronic bronchitis. Wet cough rather than sputum production was chosen as young children generally do not expectorate (although they can be observed swallowing after coughing). In an older child or adult producing sputum, this might be labelled as chronic suppurative bronchitis (152, 162), but in whatever label is chosen, all would be considered to have chronic bacterial bronchitis. As noted by the ERS task force ‘the diagnosis is confirmed by the wet cough resolving with antibiotics’. Initially, a 2-week course of antibiotics was considered to be sufficient, but this was then stretched by some to resolution after 4 weeks of treatment (165), and indeed in some cases, intravenous antibiotics are required. This is in stark contrast to the need for the two or more years of symptoms currently required to qualify for a label of ‘chronic bronchitis’ in the COPD guidelines, which as noted above is a definition intended for epidemiological studies and not clinical use. Of course, the definition is consistent with the original clinical definition of chronic bronchitis proposed by the CIBA Foundation Symposium and Medical Research Council (MRC) (139, 146). One consequence of the artificial time constraint (one or more episodes lasting at least 3 months in each of two consecutive years) for the diagnosis of chronic bronchitis is not so benign neglect rather than using aggressive approaches to treatment. Initially, the idea of PBB and hence chronic bacterial bronchitis in childhood was strongly resisted—if you do not know something exists you will not see it—but it is now widely accepted at least in tertiary care.

Reports of PBB (chronic bacterial bronchitis) such as symptoms responding to antibiotics are starting to appear in the adult literature (168–170), but the idea that these will alter our treatment of chronic bronchitis is being strongly resisted. Interestingly, the authors in one study reported that large numbers of patients who met the criteria for the study were excluded because they were already on long-term low-dose azithromycin, suggesting that clinical practice differs from the guidelines and the role of ‘prophylactic antibiotics’ is covertly embraced by many (168).

The need to respond to an antibiotic to meet the criteria for a diagnosis places bacterial pathogens clearly in the frame as the driver of symptoms. However, chronic bacterial bronchitis must start somewhere, and patients will have had a wet cough for at least 4–8 weeks (though in reality patients are usually symptomatic for much longer before a diagnosis is made) and thus will have qualified for a diagnosis of PBB long before eligible for a diagnosis of ‘chronic bronchitis’. An arbitrary cut-off of 4 or 8 weeks of coughing is simply to minimise the overuse of antibiotics for viral infections during which a wet cough is not uncommon. Delayed presentation is common amongst children with an ongoing cough with the majority being told it is ‘asthma—give her this inhaler’ or ‘it is just another virus’. It is common to find children who would meet the artificial definition required to be labelled as having ‘chronic bronchitis’ who respond to antibiotics and are thus labelled as having PBB because that is the in-vogue diagnosis. As the ERS task force noted the criteria are to be considered useful for day-to-day practice but it goes no further than that. Thus, an adult patient with ‘chronic bronchitis’ will, by definition, almost certainly have qualified for a diagnosis of PBB at some point in the evolution of the condition but the reverse is not true due to the artificial qualification criteria.

It is also of note that most, but far from all, paediatric cases commence in infancy and preschool years (157) consistent with historical reports from the pre-antibiotic era. Those studies linking early childhood respiratory illness and respiratory morbidity and mortality later in life note that it is respiratory infections in this period that link with that in late life (171–175).

The incidence of ‘PBB’ in Westernised countries also appears to be increasing given the rapid rise in publications in this area and the observations of those who have worked through this period. In part, this may be a diagnostic transfer that focuses on asthma largely to the exclusion of consideration of other airway diseases that took place in the 1980s and into the 1990s resulting in considerable misdiagnosis (both over and under). Unfortunately, this continues much as before. If you do not believe a condition exists, you will never see it. If you are taught that all chronic cough and other childhood symptoms in childhood are due to asthma and that chronic bronchitis does not exist, you will not diagnose it. Another potential factor is the progressive decline in antibiotic prescribing due to antibiotic governance having some effect on primary care (176); thus, many patients who would have inadvertently been treated at an early stage in the development of chronic bacterial bronchitis will have missed out on the ‘prophylaxis against chronic bronchitis in the next generation’. Another factor may be the effect of conjugate pneumococcal vaccines and HiB vaccines, which while suppressing the acute life-threatening illness associated with virulent strains have been predisposed to replacement with less virulent biofilm favouring strains and NTHi (177).

### ‘PBB’ (and most cases of ‘chronic bronchitis’ unrelated to smoking) is curable and early bronchiectasis is reversible

If treated aggressively with high-dose antibiotics, the vast majority can and should be cured unless there is a major underlying defect such as cystic fibrosis or antibody deficiency. In the minority of cases, this may take years of treatment dealing with relapses and involving long courses of antibiotics (155, 160). With certain underlying inherited conditions, such as CF and PCD, minimisation of damage due to recurrent or persistent bacterial bronchitis rather than cure has been the goal. With aggressive antibiotic therapy and physiotherapy together with dealing with comorbidities such as nutrition, the survival of those affected by a defect in the CFTR gene increased by many decades before the advent of disease-modifying drugs.

The vicious cycle hypothesis does suggest that in most cases in which bronchiectasis is evident on a CT scan (in adults and children), this represents medical mismanagement through failure to intervene at an appropriate time. In adult medicine, bronchiectasis is frequently stated to be irreversible. Experience in children indicates that the bronchiectasis is reversible at least in the relatively early stages. As noted above reversal of even fairly advanced bronchiectasis had also been observed in adults in the pre-antibiotic era. The lack of recent reports in adults may reflect the erroneous but almost universally held view that bronchiectasis is irreversible and a failure to be aggressive in cases in which the bronchial dilatation appears to be relatively mild. Alternatively, adults genuinely do not have the same capacity to repair damaged airways as children although studies from 70 years ago suggest that this is not the case.

### We still lack a test beyond a wet cough that gets better with antibiotics

The great challenge is still the lack of diagnostic tests. The ‘definition’ widely used for the diagnosis of ‘PBB’ is simply a description of symptoms no different to that of chronic bronchitis as outlined by the CIBA Foundation more than 60 years ago. If a patient appears to respond to antibiotics, this is used to ‘confirm’ the clinical suspicion although it should be noted that in the one randomised trial, 16% of the placebo group became cough-free in 2 weeks (as compared with only 48% in the treatment group) (178). This approach is, for example, analogous to defining, type 1 diabetes as a condition characterised by polyuria and weight loss that responds to insulin should we lack the ability to measure blood glucose. The key symptom in ‘PBB’ is a ‘wet cough’ rather than sputum production, but this is purely pragmatic as the majority of paediatric cases appear to commence in the first few years of life when children swallow rather than expectorate sputum. The ‘wet’ cough implies secretions in the airways and is not specific to PBB/Chr Br. For example, an asthmatic may have a ‘wet cough’ when the disease is poorly controlled, during an intercurrent viral infection or post an acute exacerbation. As with any symptom elicited by history, the parental reporting of wet and dry coughs is far from robust not least because a child with PBB may have a wet cough in the morning and a dry cough later in the day; hence, as always, considerable care is required when taking a history (179).

A further weakness in the ‘definition’ of PBB is that in some patients the cough does not resolve after 2 or even 4 weeks of oral antibiotics and requires a change of dose or antibiotic and occasionally intravenous antibiotics are required. This does not appear to be due to poor adherence, which appears to be a relatively uncommon problem, presumably because of the dramatic difference mothers observe in their children when on treatment. The ‘definition’ of PBB also suggests there should be an ‘absence of symptoms or signs (i.e., specific cough pointers) suggestive of other causes of wet or productive cough’ (164, 165). The specific pointers they refer to might suggest a specific aetiology (e.g., cystic fibrosis, inhaled FB, and recurrent aspiration). However, this fails to recognise that the pathology in that there is a significant chronic disturbance in the respiratory bacterial microbiome driving inflammation (persistent bacterial bronchitis) is common across the conditions and indeed they cannot be distinguished based on conventional microbiology or 16SRNA studies (180, 181).

### ‘Cinderella, you shall go to the ball’*—*chronic infection in the context of a resident microbiome

It is only recently that most respiratory physicians have finally given up the belief that the airways are sterile and the illusion that should bacteria stray below the vocal cords they are likely to cause an acute illness (182, 183). We now know that there is a very diverse pulmonary microbiome developing within hours of birth. The composition of the microbial community, as with that of the gut (from which the lungs develop embryologically) varies greatly between individuals and much is still to be learnt about how communities are maintained and evolve (163, 183–187). It has been known for many decades that ‘micro’ aspiration, especially during sleep, is normal, and this probably provides a constant supply of organisms derived from the upper airways (188–190) in addition to those inhaled. It is also of note that mucociliary clearance almost ceases during sleep and that mucociliary clearance is not a linear process in that secretions can travel in a retrograde direction entering other lobes and the contralateral lung (190). Beyond this, little is certain with some believing that the microbiome is a stable self-sustaining community while others have provided evidence that the community is largely dependent on a dynamic wash-in, wash-out environment with organisms constantly being cleared (by host phagocytes and mucociliary clearance) and replenished by further microaspiration (185–187). Amongst evidence produced to support this concept are studies showing similarity between upper airway flora and that in the conducting airways and the demonstration that the density of organisms in the airways appears to decline the further one samples from the larynx.

Given that aspiration from the upper airways seems to account for the origin of most bacteria and that their density is greatest in the more central medium and large bronchi should chronic infection develop, it is most likely to be most prominent in the earlier bronchial generation. However, such infections can also extend more peripherally through mechanisms described above, and indeed in postmortem studies, patients dying of CF biofilms in the alveoli were all too evident in the pre-very aggressive antibiotic therapy era (191). As noted by Laennec, and later by Reid in the 1950s, purulent secretions were common in the alveoli of patients dying with chronic bronchitis in the absence of bronchiectasis. More recently imaging has again shown the importance of small airway narrowing and loss in ‘COPD’ with or without bronchiectasis (192–194). The ‘overlap syndromes’ are inevitable. Recent reports have again highlighted the impact of impaired clearance and that bacterial bronchitis in ‘COPD’ with mucus plugging on CT scans is associated with increased respiratory morbidity (195) while ‘exacerbation’ is even more strongly associated with mortality (5, 196). Despite this, the GOLD guidelines suggest that these exacerbations can be treated with steroids and a 5-day course of antibiotics, which is curious given that ‘exacerbation’ in those with bronchiectasis lasts 2 weeks or more and long-term inhaled and oral antibiotics are used.

### Bacterial persistence as a cause of chronic disease—slime, IBCs, and persisters

As noted above, the last 50 years of respiratory thinking have largely ignored the progress in our understanding of microbial interactions with their host in health and disease. COPD due to smoking became the dominant concept with the idea that antibiotics had little to offer in the treatment of airway diseases (other than for the treatment of bronchiectasis and exacerbations of COPD) taking hold despite the available evidence to the contrary. This nihilism was, in large part, due to the disappointment that antibiotics were not curing patients with long-established chronic bronchitis even though they had a significant impact on quality of life. Bacteria were the cause of acute disease, and antibiotics were supposed to kill these bacteria and produce a cure.

As respiratory medicine headed in this direction, the role of ‘slime’ associated with bacteria started to become evident, and over the past 50 years, we have come to understand the role of biofilms in many chronic diseases and airway disease in particular (126–129, 191, 197–202). Indeed, bacterial biofilm communities are the default for most bacteria. In pulmonary disease, the first pathogen recognised to produce biofilms was PsA, but later, Strep Pn, Staph Aureus, NTHi, and the usual suspects were all shown to produce extracellular and, in some cases such as NTHi, intracellular biofilms. Importantly, although organisms such as NTHi and Str Pn compete when in the planktonic phase they often coexist within biofilms (148, 197, 203). In the presence of biofilms, neutrophilic inflammation is often chronic, driving symptoms and structural damage. While the neutrophils are ineffective in clearing the organisms, neutrophil nets and other neutrophil-derived materials frequently contribute to biofilm formation, particularly in those produced by NTHi (204–207).

More recently, the massive advances in sequencing have allowed investigators to start exploring the resident microbiome of the conducting airways in health and disease. The field is fraught with challenges, not least avoiding contamination from everything from the sampling process to the reagents, but they have highlighted that in patients with inflammatory airway disease, there is typically a dysbiosis but that there is no signature finding. The common characteristics of chronic bronchitis be it associated with COPD, bronchiectasis, or PBB are reduced biodiversity and an increase in abundance of some operational taxonomic units (OTUs). Interestingly, but not surprisingly, the dominant OTU determined by 16SRNA is frequently a different species from the ‘pathogen’ identified in conventional culture (163). The intersubject variations with the same disease are considerable, and, not surprisingly, the disease seems to be on a continuum with health.

What is surprising is the relative lack of discussion about the role of biofilms in permitting organisms to establish a stronghold from which they can start to occupy a disproportionate fraction of the total community and hence start to appear relatively more prevalent in 16SRNA studies. More surprising is how few respiratory physicians and indeed microbiologists appear to be exploring the implications of these concepts in chronic airway disease.

### Why we need tests beyond conventional culture

What is desperately required for clinicians to improve their care of patients who may have chronic bacterial bronchitis (a neutrophilic dominated bronchitis driven by a bacterial dysbiosis) is a test that will identify both the inflammation (this may be using metabolomic on exhaled breath) and, if possible, a robust way of identifying biofilm disease. Identification of airway neutrophilia alone is not helpful as viral respiratory infections are associated with a huge neutrophilic influx into the airways (208) and neutrophil levels can be elevated in samples from lavage from asymptomatic children (209).

Imaging has traditionally been of little value in that even established bronchiectasis frequently fails to produce significant abnormalities. In those with PBB, the changes, if present, are a generally ‘scruffyness’ due to ‘bronchial wall thickening’. With modern CT scanners and careful attention to protocols, a CT scan can be obtained for radiation doses very similar to those of a conventional CXR. In centres where this is an option, a CT scan is the radiological investigation of choice in that it more reliably identifies bronchial wall thickening and occasionally identifies unexpected pathology. It is unclear whether the observed ‘bronchial wall thickening’ is due to increased mucus or to a true thickening of the airway wall due to inflammation. While the bronchial wall thickening confirms that there is indeed some active pathological process (something which can also be inferred from the ongoing wet cough), it is not specific.

Current microbial culture approaches to identifying the drivers of chronic bacterial-driven inflammation are beset by many challenges from sampling to processing. Sputum, when available, can be helpful, but vagaries in processing can lead to significantly different results in different hands, and the results are often criticised for being contaminated by upper airway bacteria. Many argue that bronchoscopy samples (bronchial aspirates or bronchoalveolar lavage) are the gold standard, but again the evidence is that they are far from reliable and interpretation of the results is problematic other than when results agree with one's clinical suspicion!

Bronchoscopy for sample collection is rarely undertaken in adults, but it is used in paediatrics, usually when a patient relapses several times. For children, a general anaesthetic is usually required and hence is not a minor undertaking. It is well established that in symptomatic patients, the chances of growing a ‘pathogen’ increase with the number of lobes sampled even in conditions such as CF. It is also clear that the aliquots of the same sample can be sent to two different laboratories (210) (or indeed to the same laboratory) and generate significantly different results. Hence, studies suggesting sputum or cough swabs are unreliable are flawed because the ‘gold standard’ they compare is neither reproducible nor reliable. Negative sputum or BAL culture in a symptomatic patient may simply be due to failure to sample an affected lobe or may be related to processing.

Similarly, quantitative results do not appear to have a role in diagnosing chronic bacterial bronchitis, which is not surprising in that the original concept was to try and distinguish the planktonic bacterial disease from contamination and was never intended for use with biofilm-related disease. The recent North American pediatric bronchoscopy guidelines noted that quantitative cultures *migh*t be of value in identifying ventilator-associated pneumonia but have not been shown to have a role in other conditions **(**211). In a large study of children with chronic cough, the use of an arbitrary >10^4^ bacteria/ml greatly reduced the number of ‘infections’. Crucially, however, those patients in whom bacteria were cultured at lower densities were clinically indistinguishable from those with ‘infection’ based on quantitative cultures (212).

A recent study did find evidence of biofilms in many but not all of the BAL samples from children with PBB or bronchiectasis (213). These results were interpreted as suggesting that biofilms were not necessarily present in those with chronic bacterial bronchitis, but it will have been subject to many of the same issues outlined above including the low number of lobes samples and non-homogenous distribution of bacteria in the sample (214). Moreover, biofilms are relatively hard to dislodge as remaining attached to surfaces to avoid dispersal by physical forces is one of their purposes. Their definition of ‘infection’ used a quantitative level in which inevitably there would be relatively high numbers of planktonic organisms, and, of course, BAL will not recover bacteria from IBCs.

### Treatment is largely an evidence-free zone

The typical child with PBB will become cough-free after 2 weeks of treatment with a *high*-dose antibiotic such as co-amoxiclav or azithromycin (this is for its antibacterial effects not its often overhyped anti-inflammatory effects!). Typically, the improvement in the cough is slow and often with little improvement by the end of the first week (by which time, most courses in primary care and for adults have ceased) although there may be improvements such as the child appearing to have more energy, be sleeping better, and is less fractious. Of course, like many conditions, not all patients behave in the same way—some will see the cough resolve in 7 days, while others will require intravenous antibiotics to get on top of the cough. This is a common experience in the CF clinic whereby many episodes of a ‘wet cough’ will resolve with oral antibiotics although some with few respiratory problems previously may require intravenous antibiotics to treat the active bacterial bronchitis.

Relapse is common presumably due in large part to the resilience of bacteria in biofilms. IBCs, paracellular bacterial communities, and the associated persister cells (215, 216) are also important strategies used by bacteria to enable them to persist in a hostile environment such as that created by antibiotics. There is now increasing data to support our practice of using a longer course of antibiotics should the cough resolve (217, 218) (the improvement has to be dramatic and unequivocal to make the ‘diagnosis’) with typically a total of 6–8 weeks in the first instance (157, 217) (others have reported using 3 or even 6 months initially). The rationale is that this provides time for the mucosa and epithelium to recover and regenerate cilia before being recolonised. The longer courses are associated with a longer time to relapse and cure. Inevitably, there will be major intersubject variability in response to treatment.

One of the drivers for the move away from antibiotics for chronic bronchitis was concern regarding antibiotic resistance. However, a recent study (219) suggests that long-term low-dose antibiotics have less impact on bacterial resistance than a short course to treat ‘exacerbations’ as advocated by the GOLD guidelines—this may be yet another example of a well-intentioned recommendation turning out to be wrong.

It is also worth noting that studies suggest that in the vast majority of cases, a persistent dry cough not attributable to asthma will resolve by 6 months (220) but occasionally a patient with PBB will have a dry irritating cough. As with so many symptoms and signs in respiratory medicine, no one sign or symptom is unequivocally linked with a particular condition, which is not surprising given the limited range of responses of the airways.

### Recent revisions of thinking regarding ‘COPD’ have missed the elephant in the room

The lack of progress in the care of and, more importantly, the prevention of irreversible lung function impairment has raised concerns regarding the direction of travel reflected in the 2023 revision of the GOLD guidelines (221) and a recent Lancet Commission (222). However, both seemed to have again missed the elephant in the room. Infection is mentioned in both but neither gives any real credence to the importance of chronic bacterial bronchitis. Both mention TB and HIV are factors in the causation of ‘COPD’ worldwide and that early childhood respiratory illnesses are linked to increased morbidity in later life implying that they were acute hit-type events. Chronic infection receives a single mention in the Lancet publication. GOLD recommends short courses of antibiotics only for those with ‘increased purulence and volume of sputum and those being ventilated’! This is despite the fact that the same document notes that chronic bronchitis is associated with more rapid deterioration and mortality in those with ‘COPD’ and that exacerbations (acute on chronic infections) are also associated with more rapid deterioration and death. It has also been reported that the airway inflammation and associated rate of decline in lung function does not resolve in all those who give up smoking, and it has been suggested that this is due to the smoking having established its self-sustaining inflammation whereas a much more plausible explanation is that they have an associated bacterial bronchitis that would greatly benefit from treatment. Meek et al. (223) noted that those with chronic bronchitis commonly had worse symptoms and quality of life than those with chronic airflow obstruction, yet these important patient-reported outcomes are rarely discussed.

A similar cognitive dissonance occurs in the ‘field’ of ‘bronchiectasis’, where the vicious cycle hypothesis places chronic bacterial bronchitis clearly in the cycle that drives the damage that manifests as bronchiectasis. However, aggressive treatment is often initiated once the damage has been established. Where is the holistic approach to prevention? There are many people with ‘bronchiectasis’ who experience none or only mild symptoms and experience less morbidity than those with ‘chronic bronchitis’ who are not even offered a bottle of cough medicine. However, the most recent Australasian CSLD and bronchiectasis guidelines (224) do not distinguish management based on whether a radiological finding of bronchiectasis is present, treating ‘chronic suppurative lung disease’ (another name for chronic bacterial bronchitis) and ‘bronchiectasis’ as one. Management, understandably for a disease that has such a spectrum of severity, is in large part driven by the severity of symptoms. For example, should the chronic bacterial bronchitis experienced by the individual in the clinical scenario be treated to minimise the long-term damage, and would the decision be different in a non-smoker?

Chronic inflammatory airway disease may be driven by inhaled toxins, such as repeated exposure to cigarette smoke or the burning of biofuels for cooking. Equally, it can be driven by chronic bacterial bronchitis with the opportunity for initiating the vicious cycle being provided by impaired mucociliary clearance due to an extensive range of conditions including inhaled toxins, asthma, acute viral infections, or inherited conditions such as CF (see Figure 1). The vicious cycle can persist even if the enabling event resolves; for example, when the respiratory viral infection resolves, poorly controlled asthma is effectively treated asthma or when an individual gives up smoking.

### Prevention not palliation—the need for alternative or supplemental approaches

The purpose of moving bacterial infection/dysbiosis back into the centre stage is to prevent structural abnormalities from developing as far as possible rather than, as is generally the case today, waiting until a predetermined level of damage has been reached before intervening. As noted above, one of the drivers for placing chronic bacterial bronchitis in the ‘too hard box’ is the need to use long courses of antibiotics to achieve clinical improvement, which makes ‘responsible’ clinicians uncomfortable with respect to antibiotic governance—limiting its use to those with ‘bronchiectasis’ helps keep the numbers down and provides a ‘justification’. Although macrolides appear to drive greater levels of antibiotic resistance in the community (225) and individuals, they are the default for many clinicians. Evidence from ENT practice suggests that high-dose amoxicillin (90 mg/kg/day) in three divided doses is required for effective treatment of acute flare-ups of chronic secretory otitis media, yet the majority of paediatricians in many countries will use twice-daily co-amoxiclav at much lower doses for the treatment of PBB. The use of higher-dose amoxicillin with appropriate clavulanic acid is the norm, for example, in Italy (Kantar—personal communication), where it is believed that they achieve higher cure rates. Such issues should be the focus of research in both children and adults. Such high doses and long courses appeared to be required to address the challenges posed by bacterial biofilms. An acute exacerbation, often triggered by a viral infection, appears to be associated with the release of planktonic bacteria, which are much more susceptible to antibiotics and hence symptoms will start to regress towards the baseline, but eliminating infection and symptoms is a much greater challenge.

There appears to be a disconnection between the work being undertaken to understand more about the pulmonary microbiome and the mechanisms utilised by bacteria such as NTHi to effectively invade and persist in a niche normally protected from such disruptions (226) and the focus of many clinicians on lung function at the expense of exploring the role of chronic bacterial bronchitis as reflected in the GOLD guidelines. The lack of recognition that there is indeed a problem prevents the development of crucial clinical studies regarding the approach to treatment especially in adults. The necessary studies are unlikely to be funded by pharma, and since the problem is hidden in plain sight without its champions in the world of adult respiratory medicine, it remains an orphan condition.

To both improve outcomes for patients and to try and minimise antibiotic use, supplemental or alternative approaches to biofilms need to be developed. There is considerable interest in developing safe and effective biofilm disruptors (227) while others have focused on developing phages to target the bacteria. Others believe that ignoring the infection altogether and developing anti-inflammatory approaches, particularly anti-neutrophil agents, will be the way forward. Resistance to phages is likely to be a problem (228) while suppression of neutrophilic activity will have to be very tightly controlled to prevent severe side effects suggesting that a biofilm disruptor may be a better option.

However, until we have developed a position where we recognise the elephant in the room and work to place chronic bronchitis amongst the main players in the role call of airway pathology, we are unlikely to make progress.

### Summary

The airways communicate with the outside world and are vulnerable to the effects of chronic inflammation caused by chronic bacterial infections, the repeated inhalation of toxins, or the repeated aspiration of material from the upper airway (Table 2).

**Table 2:** Suggested starting point for developing cohesive nomenclature for chronic airway diseases and their consequences.

Disease	Pathology	Synonyms	Clinical impacts and consequences
Chronic bacterial bronchitis	A disturbance of airway homeostasis by persistent bacterial biofilms with neutrophil-dominated inflammation	Chronic bronchitis, CSLD, PBB, pre-bronchiectasis, symptomatic bronchiectasis, chronic endobronchial infection, pulmonary catarrh	Chronic cough and sputum production from very mild to severe *May be* associated with: • Acute exacerbations • Bronchiectasis • Accelerated decline in lung function
Toxin-induced bronchitis smoking indoor and/or outdoor pollution, recurrent aspiration	Inhaled or aspirated agents driving endobronchial inflammation		Persistent cough *May be* associated with: • Accelerated decline in lung • function • Secondary chronic bacterial bronchitis • Lung cancer (especially smoking)
Asthma	Loss of airway smooth muscle homeostasis	Reversible airway obstruction	Episodic dyspnoea or variable severity *May be* associated with: • Secondary chronic bacterial bronchitis • Accelerated decline in lung function possibly primary or more likely secondary to CBBr

*A chronic bacterial bronchitis occurs when the normal microbiome free from chronic inflammation is disrupted by biofilm forming organisms that drive a chronic neutrophilic inflammation. Any disruption to normal clearance of microbes (most commonly impaired mucociliary clearance) provides bacteria with opportunity to establish a permanent presence*.

*Inhaled toxins drive inflammation that may have little or no effect or may lead to severe airway obstruction and/or secondary bacterial bronchitis* .

*Accelerated decline in lung function should replace COPD in that intervention is required before advanced disease is established. This involves sequential lung function which should be part of an annual health review every bit as important as measuring blood pressure*.

The lower airway is a continuum from generation 0 to generation 23, and the different insults do not localise to discrete zones but produce effects from 0 to 23, and the region of maximal impact varies from patient to patient. Hence, all the conditions noted above are part of a continuum of airway disease. The last 60 years have been characterised by a focus on cigarette smoking, but there are many even in developed countries who develop COPD without smoking (228) while worldwide smoking only accounts for a proportion of those with airway disease. Addressing these is essential in promoting respiratory health, but unless we deal with the third arm, progress will be limited. The role of chronic bacterial bronchitis has been largely sidelined in large part because antibiotics did not appear to produce a cure combined with a concern about the consequences of aggressive antibiotic therapy.

The resurgence of awareness of chronic bacterial bronchitis in children and the increasing prevalence of bronchiectasis in adults provides a wake-up call to the respiratory community that the whole area of respiratory health needs to be revisited to develop objective tests and improve therapy. Those interested in micro-organisms need to be encouraged to see this as an exciting and potentially very fruitful area in which to work given the huge burden of chronic airway disease worldwide. It is essential to bring knowledge about bacterial behaviour in chronic disease, biofilms, micro-organism interactions, and their effect on the normal homeostatic processes that maintain a healthy microbiome free from chronic inflammation. Developing improved diagnostics and therapies (which will involve non-antibiotic approaches either to enhance antibiotic effectiveness or ideally effective in their own right) will go a long way to dealing with this neglected, pernicious cause of morbidity and mortality.
